# Chapter 2: Data-Driven View of Disease Biology

**DOI:** 10.1371/journal.pcbi.1002816

**Published:** 2012-12-27

**Authors:** Casey S. Greene, Olga G. Troyanskaya

**Affiliations:** Lewis-Sigler Institute for Integrative Genomics, Princeton University, Princeton, New Jersey, United States of America; Whitehead Institute, United States of America; University of Maryland, Baltimore County, United States of America

## Abstract

Modern experimental strategies often generate genome-scale measurements of human tissues or cell lines in various physiological states. Investigators often use these datasets individually to help elucidate molecular mechanisms of human diseases. Here we discuss approaches that effectively weight and integrate hundreds of heterogeneous datasets to gene-gene networks that focus on a specific process or disease. Diverse and systematic genome-scale measurements provide such approaches both a great deal of power and a number of challenges. We discuss some such challenges as well as methods to address them. We also raise important considerations for the assessment and evaluation of such approaches. When carefully applied, these integrative data-driven methods can make novel high-quality predictions that can transform our understanding of the molecular-basis of human disease.

**What to Learn in This Chapter**

- What a functional relationship network represents.
- The fundamentals of Bayesian inference for genomic data integration.
- How to build a network of functional relationships between genes using examples of functionally related genes and diverse experimental data.
- How computational scientists study disease using data driven approaches, such as integrated networks of protein-protein functional relationships.
- Strategies to assess predictions from a functional relationship network

This article is part of the “Translational Bioinformatics” collection for *PLOS Computational Biology*.

## 1. Introduction

Researchers are using genome-scale experimental methods (i.e. approaches that assay hundreds or thousands of genes at a time) to probe the molecular mechanisms of normal biological processes and disease states across systems from cell culture to human tissue samples. Data of this scale can provide a great deal of information about the process or disease of interest, the tissue of origin, and the metabolic state of the organism, among other factors. To understand biological processes on a systems level one must combine data from measurements across different molecular levels (e.g. proteomic, metabolomic, and genomic measurements) while incorporating data from diverse experiments within each individual level. An effective integrative analysis will take advantage of these data to develop a systems level understanding of diseases or tissues.

Human genome-scale experimental data include microarrays [1], [2], [3], genome-wide association studies [4], [5], and RNA interference screens [6], [7] among many other experimental designs [8]. These experiments range from those targeted towards tissue specificity [9] to those targeted towards specific diseases such as cancer [10]. The NCBI Gene Expression Omnibus (GEO) [11], a database of microarrays alone, contains over 700 human datasets collected under diverse experimental conditions encompassing more than 8000 individual arrays. The human PeptideAtlas [12], a similar resource for proteomics experiments, currently contains almost 6.7 million MS/MS spectra representing almost 84,000 non-singleton peptides across 220 samples. In addition to these high throughput experiments, there are databases of biochemical pathways [13], gene function [14], pharmacogenomics [15], and protein-protein interactions [16], [17], [18].

Integrating heterogeneous genome-scale experiments and databases is a challenging task. Beyond the straightforward concern of experimental noise in each individual dataset, integrative approaches also face particular challenges inherent to the process of unifying heterogeneous data types. Specifically we are concerned with biological and computational sources of heterogeneity. Biological heterogeneity among experiments emerges from the measurement of many different processes or the unique probing of biological systems. The source of biological material (e.g. whether experiments measure cells in culture or biopsied tissues) can also lead to systematic biological heterogeneity. Computational heterogeneity (e.g. some datasets have discrete value measurements while others are continuous) comes from the diversity of experimental platforms used to assay biological processes. Integrative approaches that bring together diverse data types and experiments must address the challenge of effectively combining these data for inference.

There are many strategies for combining these diverse and heterogeneous data. These include ridge regression [19], [20], Bayesian inference [21], [22], [23], [24], [25], expectation maximization [26], and support vector machines [27]. This chapter focuses on the strategy of Bayesian integration, which is capable of both predicting the probability of an interaction between gene pairs and providing information on the contribution of each experiment to that prediction. Bayesian integration allows for datasets to be combined based on the strength of evidence from individual datasets, which can be either learned from the data [28] or expert annotated [29]. Intuitively the Bayesian strategy works by evaluating the accuracy and coverage of each individual dataset and the relevance of each source of data to the disease or tissue of interest and using this information to weight each dataset's impact on resulting predictions. Here we discuss Bayesian methods that infer genome-scale functional relationship networks from high throughput experimental data by building on exiting gold standards. We discuss how these methods work, how to develop high quality gold standards, and how to evaluate networks of predicted functional relationships.

## 2. Combining Diverse Data Using Bayesian Inference

**Figure 1:**
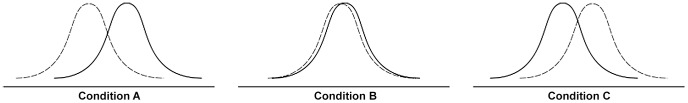
Potential distributions of experimental results obtained for datasets collected under three different conditions. The dotted line indicates the distribution of negative examples and the solid line indicates the distribution of positive examples. In condition A the positive examples more often occur to the right of the negative examples, in condition B both sets overlap, and in condition C the positive examples occur more often to the left of the negative examples.

Bayesian inference is a powerful tool that can be used to make predictions based on experimental evidence. If we want to calculate the probability that a gene of unknown function is involved in a disease, we can begin by developing a list of genes known to be involved in the disease (positive examples) and a list of genes not involved in the disease (negative examples). These positive and negative examples are termed a “gold standard” in the field of machine learning. Figure 1 shows, under three conditions, how the measurements for positive genes and negative genes are distributed in datasets measuring three hypothetical conditions. From this, we can observe that genes having a higher (more to the right) score in Condition A and a lower (more to the left) score in Condition C appear to be involved in the disease.

Bayesian inference allows us to use these distributions to quantify the probability that a gene is involved in disease given these data. Table 1 shows experimental results from Condition A where the median has been used to divide the continuous values into discrete bins.

**Table 1:** A contingency table for the experimental results for Condition A.

	Below Median	Above Median	Total
**Positive Examples**	50	150	200
**Negative Examples**	161	61	222
**Total**	211	211	422

Genes are discretized into values above or below the median. The numbers of positive and negative examples come from the gold standard. These values can be used to predict the probability that a gene with unknown status is involved in the disease.

From this contingency table we can calculate the probability that a gene *i* is involved in disease, 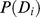, given the experimental results for gene *i*, 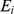. Mathematically this can be written as 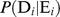. Bayes' theorem states that
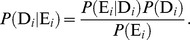



The probability that a gene is involved in disease ignoring any evidence, 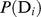, is known as the prior probability. We can conservatively estimate this as, for instance, the proportion of positive examples to the proportion of total genes. If the organism of interest has 20,000 genes, this would be
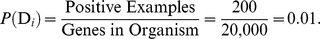



This is likely to be too conservative as it assumes that there are no unknown genes that are involved in the disease of interest. In practice, however, as evidence accumulates the impact of the prior probability on individual predictions is diminished.

With knowledge of the state of gene *i* in Condition A we can calculate 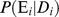. In this example, assume that the measurement for gene *i* is above the median. This probability of observing the experimental result for gene *i* given that a gene is involved in disease can be calculated as
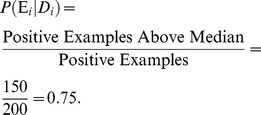



The final component of this formula is the probability of observing the experimental result that was observed for gene *i*, 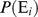. This value is the proportion of genes from the standard measured above the median to the total number of genes in the standard,
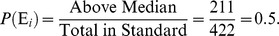



It is important to note that, if the prior is adjusted from the proportion observed in the data, 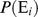 must also be adjusted to present the probability of the evidence under the new prior. With these components we can calculate the probability of disease given the experimental evidence for gene *i* as
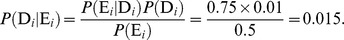



This probability is still small in large part due to our conservative prior, but by assuming that experimental results from different datasets are independent, we can perform this same calculation for gene *i* in experimental condition B using this probability as the prior, and the calculation for condition C using the probability from condition B as the prior. This procedure exploits Bayes' theorem to bring together diverse evidence sources through the common framework of probabilities.

## 3. Defining a Functional Relationship Gold Standard

Going beyond gene lists to networks of genes requires a different type of gold standard. While the inference approach described in Section 2 can be used to implicate genes in a disease or process, the specific roles of those genes remain unclear. In the strategy from Section 2, positive and negative genes make up the gold standard. By building a gold standard of positive and negative relationships, it becomes possible to predict whether or not a pair of genes interacts.

As with all machine learning strategies, the gold standard determines what type of relationship can be discovered. Here we will describe the process of building a gold standard of functional relationships, but a different standard of only physical or only metabolic interactions could be used to develop a network with those types of connections. Here we define two genes as having a functional relationship if they work together to carry out a biological process (e.g. a KEGG pathway) that can be assayed by definitive experimental follow-up. This definition allows us to capture diverse types of relationships, while discovering relationships suitable for biological follow-up. The Gene Ontology's biological process ontology provides annotations of genes to process, but includes both very broad and very narrow processes. Two examples of broad terms would be “biological regulation” and “response to stimulus.” Two examples of narrow terms would be “positive regulation of cell growth involved in cardiac muscle cell development” and “cell-matrix adhesion involved in tangential migration using cell-cell interactions.” The broad terms are not specific enough to provide a meaningful gold standard, while the narrow terms have too few annotations to provide sufficient examples of known relationships.

To address this shortcoming, Myers et al. [30] used a panel of experts to select terms from the biological process ontology that were appropriate for confirmation or refutation through laboratory experiments such as “response to DNA damage stimulus” and “aldehyde metabolism.” These terms can be downloaded and used to build a positive functional relationship standard. Gene pairs where both pairs share one of these terms can be considered to have a functional relationship. Gene pairs which do not share an annotation are of unknown status. For Bayesian inference we must also have a negative standard. One potential way to develop a negative standard would be to randomly select pairs of genes. This assumes that most pairs of genes do not interact.

It is possible to add additional high quality experimentally annotated relationships to these standards from other databases. Databases like KEGG [13], Reactome [31], and HPRD [32] have previously been used to identify additional functional relationships [33]. The positive and negative relationships from the standard determine the type of relationship that will be predicted by the Bayesian integration. Here we use functional relationships, but a gold standard built strictly from physical protein-protein interactions will infer only physical interactions relationships between genes.

## 4. Building a Network of Functionally Related Genes

Given a gold standard of gene-gene relationships, the probability that two genes of unknown status have a relationship can be calculated from diverse data using Bayesian inference. The process is similar to the integration process described for single-gene prediction, but there are differences. For each dataset, appropriate scores for each gene pair must be calculated. Furthermore, these scores should not require any manual intervention or adjustment that would make an analysis of hundreds or thousands of datasets time consuming. For datasets that are naturally made up of pair-wise scores such as yeast two-hybrid assays, this task is straightforward. For datasets made up of individual gene measurements, such as microarray experiments, a useful measure must be found.

One measure that can provide pair-wise scores across arrays is correlation. Correlation quantifies the amount that two genes vary together and can be a useful indicator of functional relationships. Comparing correlation across datasets in a regular manner is difficult however, because datasets may display more or less correlation based on both true biology (e.g. under some conditions more genes vary together) or experimental error (e.g. systematic biases due to hybridization conditions) and the variance of gene-wise correlations would vary based on these dataset dependent effects. Fisher's z-transform provides a means to convert these correlation coefficients (*r*) to z-scores by calculating z as
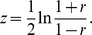



These z-scores provide a familiar framework to work with correlation and allow correlation measures between genes to be compared across datasets. It is then possible to categorize genes pairs as negatively correlated, uncorrelated, or positively correlated based on whether their z-score is less than, approximately equal to, or greater than zero.

These pairs can then be used as evidence in an integration. In the single gene situation, we were interested in 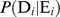, or the probability of gene 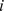 causing disease given its evidence. Here we are interested in the probability of a functional relationship between genes *i* and *j*, 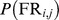, given some pair-wise evidence (e.g. correlation), 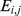. As in the single gene situation, this can be calculated with
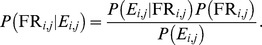



Like before, a contingency table is used. The difference in this situation is that the table is based on pair-wise gene measures instead of measurements for individual genes. This process, when used to calculate pair-wise probabilities of functional relationships for all of the genes in the genome of interest, results in a functional relationship network for the organism of interest.

**Figure 2:**
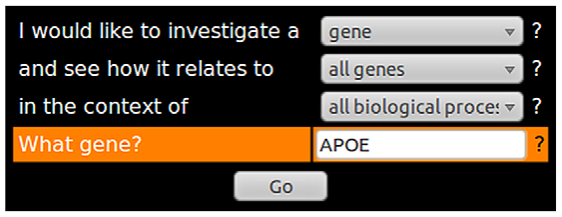
An example of querying HEFalMp for the role of APOE across all biological processes (http://hefalmp.princeton.edu/).

**Figure 3:**
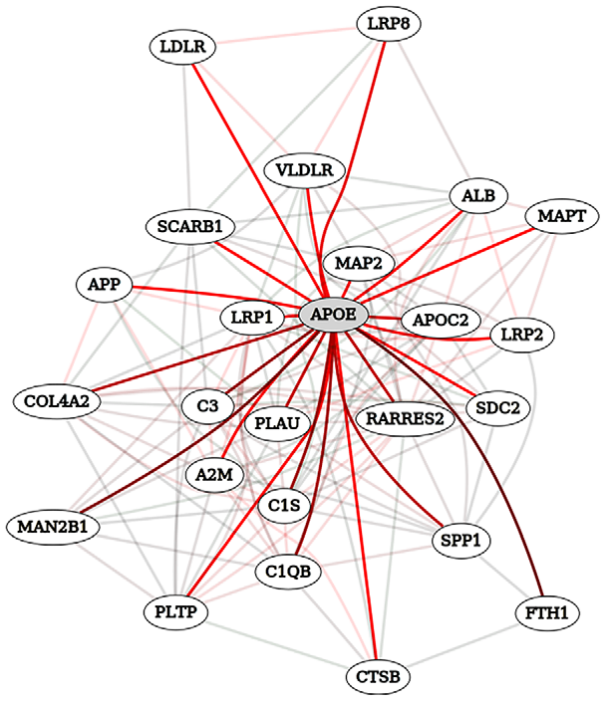
The result of querying HEFalMp for the role of APOE across all biological processes. Red links indicate that there is a high probability of a functional relationship between the two genes.

Huttenhower et al. [33] performed Bayesian integration and prediction using human gold standards and datasets. This tool allows users to query the network and also displays what datasets contribute to the relationships predicted from the integrated approach. As an example we can query HEFalMp to find out how the APOE protein relates to all genes across all biological processes as shown in Figure 2. The result is shown in Figure 3. The red links indicate that there is a high probability of a functional relationship between the two genes and green links indicate a low probability. Black links indicate a probability of approximately 0.5.

**Figure 4:**
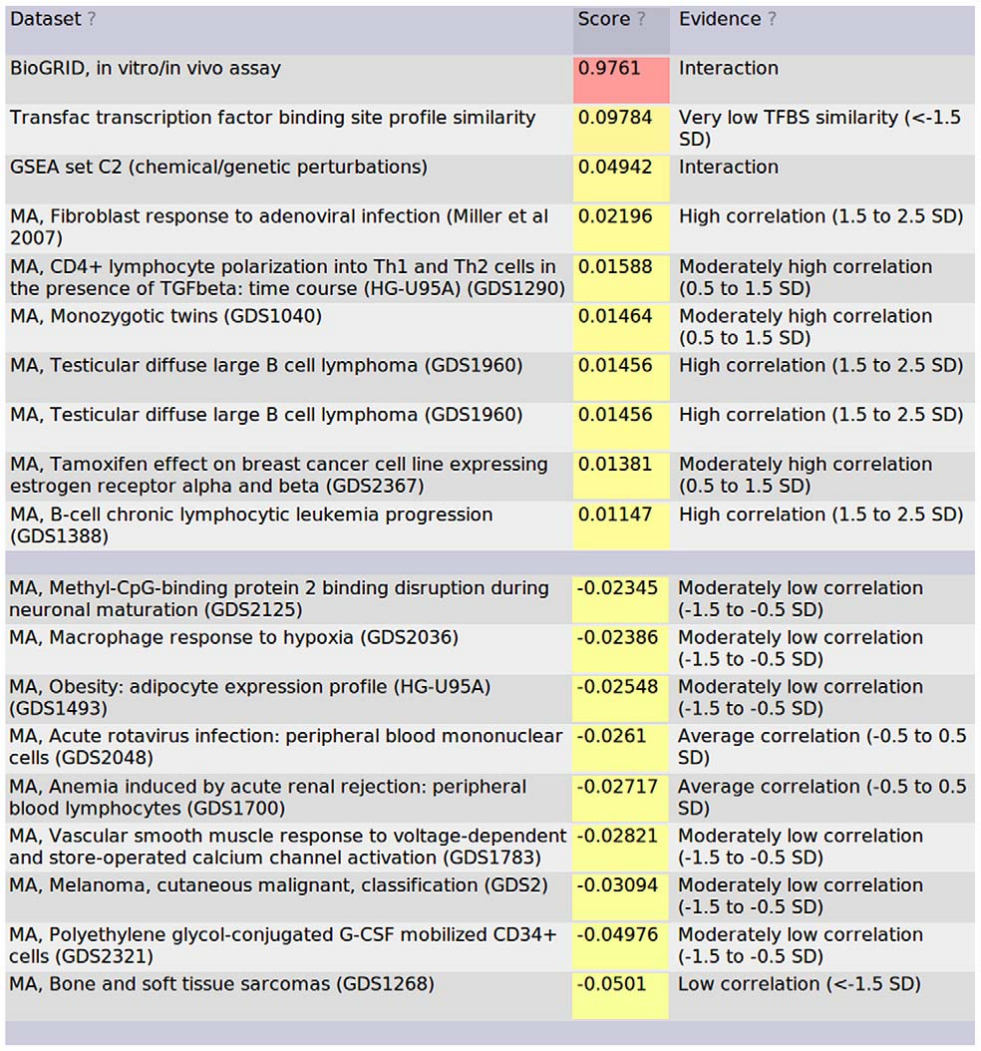
The highest and lowest contributing datasets for the pair of APOE and PLTP are shown (http://hefalmp.princeton.edu/gene/one_specific_gene/18543?argument=21697&context=0). These contributions are based on how well the bin containing the queried gene pair separated known positive functional relationships from known negative functional relationships.

The probability of a functional relationship between any pair of genes is calculated as described previously. As such, this probability is dependent on evidence from each individual dataset. By clicking on a link, the contributions for each dataset towards that gene pair are provided as shown in Figure 4 for APOE and PLTP. This figure indicates the value of including high quality databases such as BioGRID as input data. While the microarray datasets are informative, in this case the three highest weighted datasets were non-microarray data sources.

**Figure 5:**
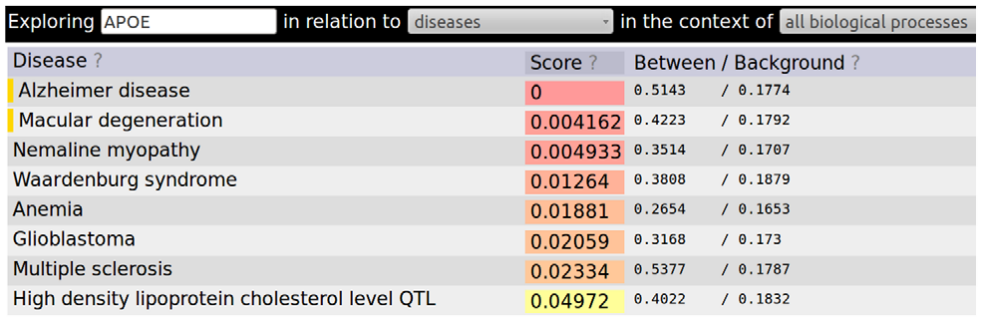
The diseases that are significantly connected to APOE through the guilt by association strategy used in HEFalMp. Alzheimer disease and Macular degeneration are both annotated to the disease in OMIM as noted by the gold bars to the left of the disease (http://hefalmp.princeton.edu/gene/diseases?context=0&name=APOE). The other diseases are implicated by APOE's functional relationships to genes annotated to that disease in OMIM.

**Figure 6:**
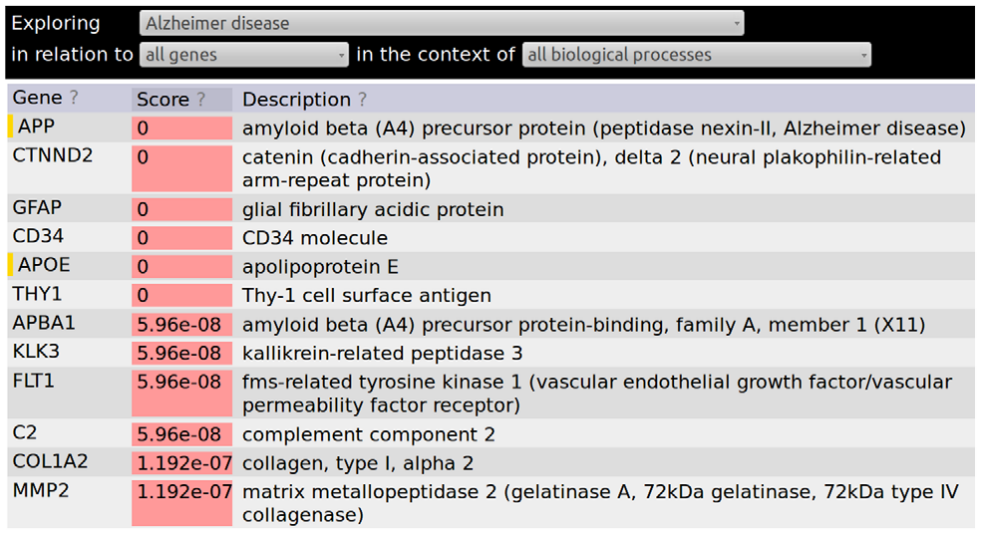
The genes that are most significantly connected to Alzheimer disease genes using the HEFalMp network and OMIM disease gene annotations (http://hefalmp.princeton.edu/disease/all_genes/55?context=0). The gold bars to the left of APP and APOE indicate that both genes were annotated Alzheimer disease according to OMIM.

These functional relationships can then be used to connect genes to diseases through guilt by association approaches. Guilt by association approaches work by finding genes or diseases that are highly connected to query genes. How exactly this is done depends on the underlying network, the size and type of the query sets, whether or not the task must be done in real time. An example approach would be to consider as positives only relationships with a probability from the inference stage of greater than 0.9. A Fisher's exact test p-value [34] can then be calculated using the counts of genes connected to the query, the number of genes connected to the query and annotated to the disease of interest, as well as the total number of genes in the network and the number of those genes annotated to the disease [34]. The approach used by the HEFalMp online tool is more complicated because the network-specific calculations must be done in real time for the web interface. Figure 5 shows diseases significantly associated with the APOE protein through the HEFalMp online tool, while the procedure used to generate the results for Figure 6 flips the analysis and shows genes significantly associated with Alzheimer disease based on their connectedness to genes annotated to this disease in OMIM [35].

## 5. Evaluating Functional Relationship Networks

After performing a Bayesian integration it is appropriate to assess the quality of the inference approach. One straightforward way to evaluate the network would be measure the concordance of the gold standard and predictions from the network. This is easily done by ordering gene pairs by their probabilities in the network from highest to lowest. For each gene pair in the gold standard, the true positive rate (TPR) to that point can be calculated as
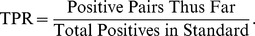



The false positive rate (FPR) can be calculated with the same values for negative pairs. These values can then be plotted with FPR on the horizontal axis and TPR on the vertical access. This provides one type of receiver-operator characteristic (ROC) curve which can be used to assess the quality of predictions from the network. The area under this curve (AUC) summarizes to a single number the quality of predictions.

Unfortunately this approach to evaluation uses the same evaluation standard as the gold standard used for learning and therefore it tests the ability of the inference approach to match the gold standard, and not its ability to make new predictions. One way to avoid this circularity is to hold a group of genes out of the gold standard during the integration process. Connections between these held out genes can then be used after the networks are generated to assess the quality of predictions from the network (in this case the concordance between the predictions and the known relationship status of the held out genes are used). While the holdout approach is effective for large gold standards, when gold standards are small this can result in too few known relationships for assessment of the network. This assessment problem can be alleviated at the cost of computation time by using a cross-validation approach. With cross-validation, the gene sets are divided up into groups. Like the hold-out approach, all but one group is used to train the network while the evaluation is performed on the left out group. In contrast to the hold-out approach, the process of training and evaluation is performed iteratively with each group of genes being evaluated, but like the hold-out approach, only the predictions generated on held out genes are used for evaluation.

When standards are incomplete, existing literature can also be used for evaluation. This can be incorporated in a number of ways. One way is to use a blind literature evaluation. Pairs predicted with high probability or genes highly connected to members of the standard can be selected for follow-up. These are combined with randomly selected genes to create a gene list for evaluation. Literature evidence for genes on this list can be assessed, and a comparison can be performed for genes selected from the network and genes selected randomly. If the proportion of literature based positives of genes or pairs selected from the network is substantially higher than those selected randomly, this provides evidence that the network recapitulates true biology.

Fundamentally the goal of this data driven functional genomics strategy is to create a network of predictions useful for designing biological experiments [36]. If these predictions lead to a higher success rate in molecular biology experiments, an integrative analysis can dramatically lower the cost per discovery. Hibbs et al. [37] used a data driven approach to direct experimental biology and found that computational predictions could be experimentally validated at a substantially higher rate than randomly selected genes. Furthermore, those genes that were found by computational methods were more likely to exhibit a subtle phenotype than the genes already known to be involved. This study provides evidence that computational predictions combined with experimental science can lower the cost of experimental discoveries while finding subtle phenotypes that high throughput experimental designs may miss.

## 6. Summary

Data driven functional genomics strategies combine methods from statistics and computer science to integrate diverse experimental data for the purpose of making novel biological predictions. By bringing diverse data together, these methods are capable of discovering patterns of biological relevance not well characterized in individual studies [38]. Furthermore, because these methods rely on existing data, they can be used to efficiently direct definitive low throughput experimental studies in a cost effective manner [37], [39].

Integrative data driven approaches are often compared to publicly available databases of knowledge or experiments or to the statistical analysis of results from individual high throughput experiments, but they are distinct from both of these. Databases generated by literature curation are by their nature not well suited to the discovery of new knowledge and databases of experimental results require researchers to know *a priori* which datasets are relevant to the biological question of interest. Integrative data driven approaches combine high throughput experiments and databases of diverse types and in so doing can make predictions beyond those discovered using single data sources.

The flexibility of the data driven approach also gives rise to its greatest challenge. This strategy relies upon gold standards that are a representation of high quality current knowledge. When these standards are of high quality and appropriate to the biological question of interest, the resulting answers are likely to be useful. If the standards are of lower quality, the utility of the predictions will be lessened. In many cases the gold standard quality is the critical determinant of success for these algorithms. With careful use, these methods can generate predictions capable of efficiently directing experimental biology [37], [40].

## 7. Exercises

**Figure 7:**
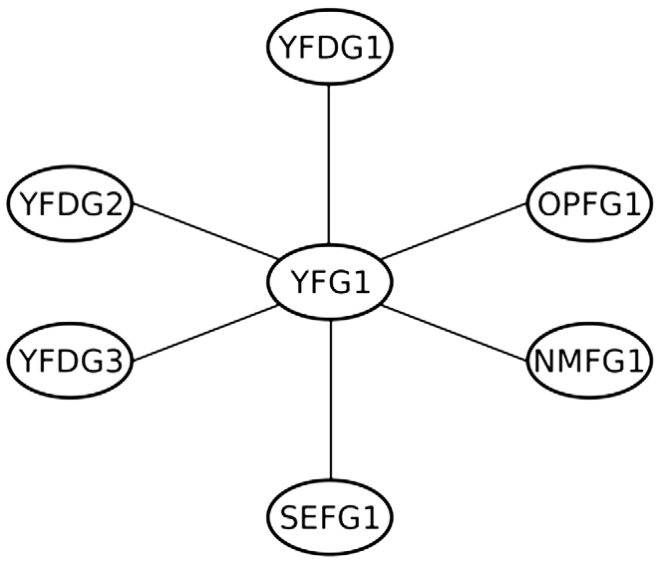
The functional relationship network discovered by a data driven integration for the YFG gene in YFO.

1. All proteins connected to the protein Your Favorite Gene (YFG) in the functional relationship network of Your Favorite Organism (YFO) are shown in Figure 7. Three of them are known to be associated with Your Favorite Disease (YFD). These genes are YFDG1, YFDG2, and YFDG3. YFD has six genes annotated to it among the 100 genes present in YFO. Using a Fisher's exact test to evaluate guilt by association, is YFG significantly associated with YFD (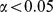)?
2. Does the gene expression dataset described by the contingency table in Table 2 provide any information about whether or not the genes YFG and MFG are likely to have a functional relationship if they are uncorrelated in this dataset? What if they are negatively correlated?
3. Using the contingency tables from Tables 2 and 3 and the knowledge that 20% of gene-pairs in the organism of interest have a functional relationship, what is the probability that genes YFG and MFG have a functional relationship if they are positively correlated in the experiment that Table 2 is derived from and physically interacting in the database from which Table 3 is derived?
4. What is the major difference between databases and integrative data driven approaches?

Answers to the Exercises can be found in Text S1.

**Table 2:** A contingency table for gene-pairs based on correlation in a gene expression dataset.

	Negatively Correlated	Uncorrelated	Positively Correlated
**Known Positive Relationships**	20	30	50
**Known Negative Relationships**	400	300	200

**Table 3:** A contingency table for gene-pairs based on a database of physical interactions.

	Not Physically Interacting	Physically Interacting
**Known Positive Relationships**	10	90
**Known Negative Relationships**	900	100

**Further Reading**

- Kanehisa M, Bork P (2003) Bioinformatics in the post-sequence era. Nat Genet 33 Suppl: 305–310.

**Glossary**

- Functional Relationship: The type of interaction that two genes have if they participate in the same biological process.
- Gold Standard: A set of genes or gene-pairs with a known status (positive or negative) in the tissue, process, disease, or phenotype of interest.
- Hypergeometric/Fisher's Exact Test: A test of independence appropriate for categorical count data when the number of items in each cell is small.

## Supporting Information

Text S1 Answers to Exercises
(DOCX)
