# Supplementary material for: Chapter 2: Data-Driven View of Disease Biology
Source: PLoS Comput Biol. 2012 Dec 27;8(12):e1002816. doi: 10.1371/journal.pcbi.1002816 (PMC3531282; doi:10.1371/journal.pcbi.1002816)
Supplement: Text S1 — Answers to Exercises (DOCX) [file pcbi.1002816.s001.docx]

## Text S1: Exercise Solutions

1. All proteins connected to the protein Your Favorite Gene (YFG) in the functional relationship network of Your Favorite Organism (YFO) are shown in Figure 7. Three of them are known to be associated with Your Favorite Disease (YFD). These genes are YFDG1, YFDG2, and YFDG3. YFD has six genes annotated to it among the 100 genes present in YFO. Using a Fisher’s exact test to evaluate guilt by association, is YFG significantly associated with YFD ($\alpha<0.05)$?

First we build a contingency table for this situation:

|  | YFG-Connected | YFG-Unconnected |
| --- | --- | --- |
| Disease-annotated | 3 | 3 |
| Not disease-annotated | 3 | 91 |

We can put this table into R (<http://r-project.org>) with:

**contingency <- matrix(data=c(3, 3, 3, 91), nrow=2, ncol=2)**

and run the Fisher’s exact test with **fisher.test(contingency)**

This produces:

**Fisher's Exact Test for Count Data**

**data: contingency**

**p-value = 0.002304**

**alternative hypothesis: true odds ratio is not equal to 1**

**95 percent confidence interval:**

**2.612837 318.177298**

**sample estimates:**

**odds ratio**

**27.26141**

Therefore, we would say that there is a significant association because p < $\alpha$.

1. Does the gene expression dataset described by the contingency table in Table 2 provide any information about whether or not the genes YFG and MFG are likely to have a functional relationship if they are uncorrelated in this dataset? What if they are negatively correlated?

First we can calculate the prior probability of a functional relationship using the gold standard fraction observed in this dataset.

$$P\left( \mathrm{FR}_{i,j} \right)= \frac{Positive Relationships}{Total in Standard}= \frac{100}{1000}=0.1$$

Then we can calculate the probability of a functional relationship given that a pair is uncorrelated:

$$P\left( \mathrm{FR}_{yfg,mfg}|Uncorrelated \right)= \frac{P\left( Uncorrelated|\mathrm{FR} \right)}{P\left( Uncorrelated \right)}* P\left( \mathrm{FR}_{i,j} \right)= \frac{\left( \frac{30}{100} \right)}{\left( \frac{30}{100}*0.1 \right)+\left( \frac{300}{900}*(1-0.1) \right)}*0.1=0.091$$

Thus the uncorrelated state of our pair in this dataset has told us something, the genes are slightly less likely to be involved in a functional relationship than a randomly chosen gene pair (i.e. our dataset-chosen prior of 0.1). Note that even though we are using the dataset prior, the adjustment for different priors (0.1 and 1-0.1) is shown for clarity and continuity for exercise 3.

The calculation for negatively correlated is of the same format:

$$P\left( \mathrm{FR}_{yfg,mfg}|Negatively Correlated \right)= \frac{\left( \frac{20}{100} \right)}{\left( \frac{20}{100}*0.1 \right)+ \left( \frac{400}{900}*(1-0.1) \right)}*0.1=0.048$$

Thus the negatively correlated state of our pair in this dataset has told us something. These genes are less likely to be involved in a functional relationship than a randomly chosen gene pair.

1. Using the contingency tables from Tables 2 and 3 and the knowledge that 20% of gene-pairs in the organism of interest have a functional relationship, what is the probability that genes YFG and MFG have a functional relationship if they are positively correlated in the experiment that Table 2 is derived from and physically interacting in the database from which Table 3 is derived?

This formula follows the same format as the prior question, but both evidence sources are combined (for both the numerator and denominator) and we are now given the prior probability of a functional relationship by:

$$P\left( \mathrm{FR}_{yfg,mfg} \right)= \frac{\left( \frac{50}{100} \right)*\left( \frac{90}{100} \right)}{\left( \frac{50}{100}*\frac{90}{100}*0.2 \right)+\left( \frac{200}{900}*\frac{100}{1000}*(1-0.2) \right)}*0.2=0.835$$

It is important to note that this is a situation where $P(E)$ had to be adjusted to account for a prior different than the priors in the datasets.

1. What is the major difference between databases and integrative data driven approaches?

Databases provide a catalog of interactions (physical, genetic, etc.) that have been observed by researchers. Integrative approaches combine these known interactions with genome-scale observations of the system (e.g. gene-expression, protein-protein interaction) to make new predictions of interactions.
